# Mental Health Risk Factors and Coping Strategies among Students in Asia Pacific during COVID-19 Pandemic—A Scoping Review

**DOI:** 10.3390/ijerph19158894

**Published:** 2022-07-22

**Authors:** Wandeep Kaur, Vimala Balakrishnan, Yoke Yong Chen, Jeyarani Periasamy

**Affiliations:** 1 Faculty of Information Science & Technology, Universiti Kebangsaan Malaysia, Bangi 43600, Malaysia; 2 Faculty of Computer Science and Information Technology, Universiti Malaya, Kuala Lumpur 50603, Malaysia; vimala.balakrishnan@um.edu.my; 3 Department of Psychological Medicine, Faculty of Medicine and Health Science, Universiti Malaysia Sarawak, Kota Samarahan 94300, Malaysia; yychen@unimas.my; 4 Faculty of Data Science and Information Technology, INTI International University, Nilai 71800, Malaysia; jeyarai.periasamy@newinti.edu.my

**Keywords:** mental health, depression, stress, anxiety, students, higher education, COVID-19, Asia Pacific

## Abstract

The impact of COVID-19 has forced higher education institutes to go into lockdown in order to curb the situation. This sudden change caused students within the institutions to forgo traditional face to face classroom settings and to attend immediate online classes. This review aims to summarize the evidence of the social demographic mental health impacts of the COVID-19 pandemic on students in higher education institutes within the Asia Pacific region and identify the coping mechanisms adopted during these times. A systematic literature search was conducted using three databases (PubMed, Google Scholar, and Scopus), out of which 64 studies met the inclusion/exclusion criteria. The findings revealed that the social demographic groups most at risk were female students, those who were in the final years of their studies (i.e., students who were almost graduating), and postgraduate students as well as students studying medical fields (nursing, dental, medicine, health sciences etc.). The majority of the studies identified that students were relying on mobile devices and extended screen time to cope with the pandemic. Having proper social support, be it through a network of friends or positive family cohesion, can be a good buffer against the mental impacts of COVID-19. Students in higher education institutes are at risk of mental consequences due to COVID-19. By reducing their screen time, finding a healthier coping system, increasing the availability of support within the family and community, as well as actively engaging in beneficial activities students may be able to alleviate general negative emotions, specifically during the pandemic.

## 1. Introduction

The Coronavirus disease (COVID-19) pandemic has spread to 198 countries, with approximately 555 million confirmed cases and 6.35 million deaths globally as of 12 July 2022. The 2019 Coronavirus (COVID-19) pandemic led governments across the globe to enforce movement restrictions and social segregation orders across countries leaving only essential services open. This also included suspending the traditional classroom setting in all educational institutes and shifting to remote methods of teaching as well as assessment. The continuous spread of the virus, travel restrictions, and the indefinite closure of educational institutions are reported to have had a substantial impact on students’ education, social lives, and mental health [1,2,3].

It is estimated that 8% to 71% of students in higher education institutions across the globe have suffered from acute stress disorder, depression, and/or anxiety symptoms during the COVID-19 pandemic [1,2,3,4,5,6]. The large discrepancy in reported prevalence may be due to the different questionnaires and methodologies used (for example, [4,5,6]). The time of assessment may contribute to the difference in prevalence reported. This is because most of the psychological sequelae, especially those of disaster-related events, only occur a period of time after a crisis, for example, the Wenchuan earthquake survivors exhibited suicide ideation mostly six months after the crisis [7].

Growing acknowledgement of the psychiatric implications of COVID-19 may be due to the social impact of the current pandemic and government policies including social distancing measures, travel restrictions, personal hygiene etiquette, quarantine, and business-related restrictions [8,9]. The whole community, including those infected or non-infected, were susceptible to the sequelae of such policies and/or movement, such as higher levels of anxiety, stigma, unemployment rates, and financial difficulties [10].

A study on Chinese students found that 25% of them were experiencing some level of anxiety due to the changes [11]. Students were forced to live away from their parents during this period of time causing their psychological wellbeing to deteriorate [11]. Various studies carried out this past year have gone on to show that female students and those who were living in rural areas in particular, manifested higher levels of stress and depression as they had a fear of losing out in their academic year [3,12]. A study by Szuster [13], also highlighted the plight of young women whose depressive symptoms and fear intensified with subsequent lockdowns. Furthermore, the overnight change to online learning platforms coupled with a lack of information technology knowledge amongst non-technical educators also caused frustration among students [12]. These are some of the reasons why this current review aimed to focus on students in higher education institutions. The novelty of this scoping review is in summarizing all works that have been published during the very first year of COVID-19 pandemic lockdowns, specifically where the demographic was students in higher education institutes within the Asia Pacific region. In identifying the risk factors, this study found that students adopted various coping mechanisms to deal with the pandemic to the best of their ability.

Evidence shows that depression, stress, and anxiety prevalence increased as time progressed [14,15,16]. As an example, Wong [16] assessed the status of mental health among Malaysian people using several timelines ranging from May 2020 to September 2020 with results indicating that depression prevalence increased dramatically as the pandemic progressed. Therefore, there is a need to update and extend the literature to encompass more recent studies, particularly a year after the beginning of the COVID-19 pandemic across the affected population. Recently published systematic review studies have looked into the impact of COVID-19 on the mental health of children, adolescents, and college students [17,18]. Elharake [17] conducted a review study on children, adolescents, and college students, however, it focused on the impact of COVID-19 on the mental health of college students where they discussed the prevalence of various mental health conditions such as depression, PTSD, stress, and anxiety. Furthermore, the number of papers reviewed in that study was only 16, while 64 articles were reviewed for this study making this study more comprehensive. Other review studies are country specific [19,20,21]; and some review studies looked at comparing mental health prevalence pre, during and post pandemic [22,23,24]. This systematic review aims to specifically (a) identify risk factors that contribute towards depression, anxiety, and stress among the higher education student population in Asia Pacific countries, (b) identify coping strategies adopted by students to battle mental health issues that arose during this period. To the best of our knowledge, our systematic review is the first to examine the risk factors as well as the strategies adopted by students in the Asia Pacific region to cope with depression, stress, and anxiety during the COVID-19 pandemic.

## 2. Methodology

This is a sub study of a wider review study that was undertaken utilizing a flexible and iterative methodology to address a broad variety of research [25,26]. A five-stage framework [27,28] was used and reported in congruity with the regulation in the Preferred Reporting Items for Systematic Reviews and Meta-Analyses (PRISMA) extension for scoping reviews [29]. Each step will be explained in detail as follows.

STEP 1: Identifying Research Questions

Two research questions were identified for this scoping review study:

(a) What are the socio-demographic factors impacting the mental health of students in the Asia Pacific region during the COVID-19 pandemic?
(b) What are the coping strategies adopted by students in the Asia Pacific region during the COVID-19 pandemic to overcome their depression, stress, and anxiety?

STEP 2: Searching for Relevant Studies

Studies identified for this scoping review were part of a larger systematic review study identifying prevalence of depression within the Asia Pacific region during the COVID-19 pandemic. The interest of this review was to look into published work relevant to the Asia Pacific regions and understand the impact COVID-19 had, particularly on students at higher learning institutes within this region. Therefore, scholarly publications were sought within the domain of mental health in the region of Asia Pacific. Three electronic databases (PubMed, Google Scholar, and Scopus) were used to search for articles that had been published from January 2020 to March 2021. The search method employed several keywords using boolean and wildcard operators including “mental health and pandemic”, “mental health and C*”, “mental health and outbreak” and “mental health and epidemic”. Additionally, specific keywords such as “mental health” with mental issues, stress*, depression, anxiety, emotion* and psycho* were also used resulting in 1979 articles extracted in total. EndNote was used to manage the review process as well as to eliminate any possible duplication.

STEP 3: Studies Selection

During this phase, an initial screening was conducted by drawing out the inclusion and exclusion criteria where papers that fulfilled the following were removed:

- Pre-prints or pre-approved journals (i.e., unpublished journals as of the date of extraction);
- Non-English articles;
- Review papers/Opinion papers/Letters to Editors/Short communication etc. In short, papers that did not provide any research data related to mental health;
- Studies involving clinical trials as well as pre-existing psychological disorders or mental illness prior to the pandemic.

The research question of this scoping review is to identify the factors affecting the mental health of students resulting from a direct impact of COVID-19. Therefore, papers that did not fulfil the objective of this research question needed to be removed. Papers that met the eligibility criteria include:

- Papers where the cohort under study was strictly students in higher learning institutes;
- Articles that examine the mental health of students of any age, any educational background (medical, engineering, law, management etc.) and any level (undergraduate or postgraduate) during the time of COVID-19 within Asia Pacific countries;
- Articles that pay close attention to the mental health consequences of the COVID-19 pandemic.

Recent systematic reviews related to mental health during COVID-19 were also checked upon to ensure that a study of a similar kind had not been carried out. Recent systematic studies focused their efforts on frontliners and healthcare workers [30,31,32]. The review done on adolescents looked into the prevalence of depression and anxiety within the populations of adolescents (straight, bisexual, transgenders etc.) [33]. However, this paper is looking to bridge the gap within the research by looking into the mental health impacts from the students’ perspective within the Asia Pacific region.

STEP 4: Charting the Data

Studies that were screened from the previous step were then individually looked into where the study characteristics as well as the narrative synthesis was deducted. Three reviewers looked into the studies independently in order to identify and screen studies that fulfilled the two research questions in Step 1.

STEP 5: Collating, Summarizing, and Reporting the Results

An iterative approach was adopted to report the findings. Each journal identified in Steps 2 and 3 went through several cycles of screening keeping the objective of answering the research question in mind before a finalized list of articles was agreed upon.

## 3. Results

**Figure 1:**
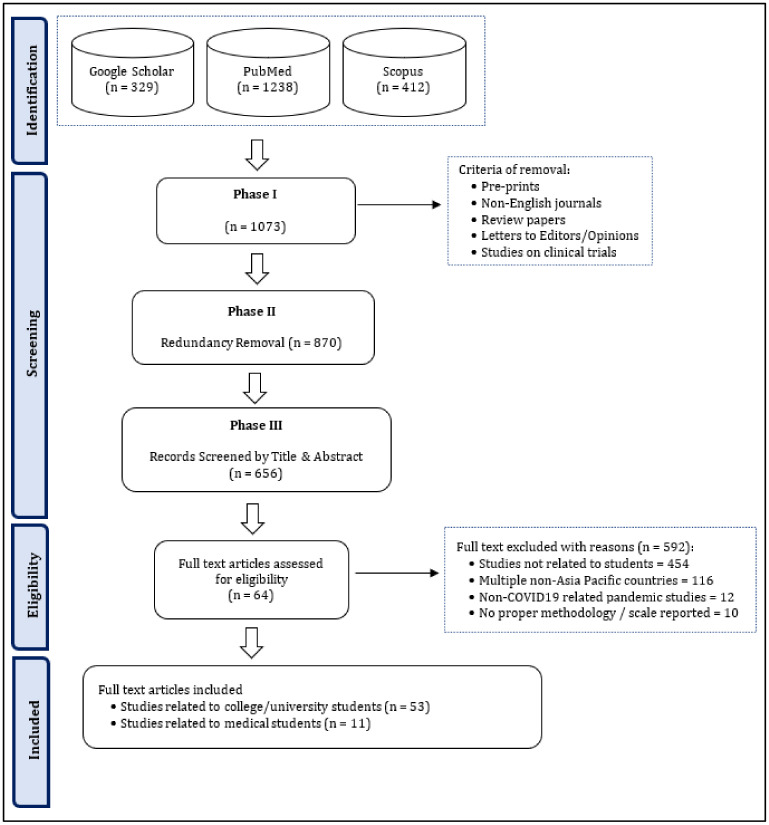
Flow diagram that describes the flow of information through the review’s study search and selection.

The initial search yielded 1929 papers which was reduced to 1073 after checking for the first stage of inclusion and exclusion criteria as explained in Step 3 and was further reduced to 870 papers after deduplication. Sixty four articles were distilled after full-text review (Figure 1).

### 3.1. Description Data

Table 1 displays the characteristics of the included articles. More than half (*n* = 38, 59%) of the studies were published in 2020. The largest portion of studies were from East Asia (*n* = 28, 44%), i.e., mostly China (*n* = 24), followed by South Asia (*n* = 17, 27%), and Bangladesh (*n* = 10), and North America (*n* = 14, 22%), i.e., mostly in the United States of America (*n* = 13). The majority of the studies (*n* = 53, 83%) were conducted on university or college students from non-medical backgrounds. Overall, the most common form of study design used was cross sectional (*n* = 51, 80%), and the commonly used sample size was between 100–999 (*n* = 38, 59%). The general anxiety disorder (GAD-7) and Patient Health Questionnaire (PHQ) scale was the most frequently used scale (*n* = 18, 28%). Among the other scales used were Zhung’s Self Rating Anxiety Scale [34,35], Centre for Epidemiological Studies Depression (CESD) [36,37,38] and the Perceived Stress Scale [39,40,41] etc.

### 3.2. Social Demographic Factors Impacting Mental Health of Students

In the 64 papers included in this scoping review study, the factor that affected students’ mental health the most was gender (*n* = 22) where the female gender was found to be impacted more than the male. Students who were in their final year of studies and postgraduate students who had almost completed their studies were affected more than students who were still in the midst of their academic years (*n* = 8). Other factors identified were age (*n* = 11), economic and financial stressors (*n* = 11), geographical location of students i.e., urban or rural (*n* = 9), and current living conditions i.e., living alone or with family and friends (*n* = 10). Five studies also identified race and ethnicity as being a factor. For example, Charles [39] found that white students were mentally more affected by the pandemic compared to African Americans contradicting Hoyts [40], who found that African American students reported marginally higher anxiety compared to white and Asian students. In a mixed region study of Thai, Indonesian and Taiwanese students, it was found that Thai students reported the highest levels of anxiety [42].

Studies have been categorized according to the mental health outcomes identified: depression-like symptoms, anxiety-like symptoms, stress and other psychological distress (Table 2 and Table S1) while acknowledging an overlap between these categories.

#### 3.2.1. Depressive Symptoms

Thirty four studies looked into depressive symptoms amongst students. The female gender was most associated with depressive symptoms [1,2,4,37,43,44,45,46,47,48], however, one study did not report any significant difference between genders in their study [36]. Ten studies reported depressive symptoms amongst students who were in the final stages of their academic years [1,4,36,44,48,49,50,51,52]. The estimated depression prevalence from within the 64 studies was between 4.44% and 72% (95% CI). The range difference includes studies that have identified mild depression to severe depression.

#### 3.2.2. Anxiety Symptoms

Thirty nine studies looked into anxiety symptoms amongst students and the estimated anxiety prevalence recorded was between 0.9% and 58.7% (95% CI). The range difference includes studies that have identified mild anxiety to severe anxiety. Similar to depressive symptoms, the female gender [1,2,34,35,36,40,44,45,46,47,48,53] and year of study [1,34,35,36,44,48,49,50,51,52] were associated with elevated levels of anxiety among students. However, all studies using medical field students [1,11,54,55,56,57] reported anxiety symptoms as students were unable to complete their clinical training due to the pandemic situation.

#### 3.2.3. Stress

Thirteen out of 16 studies that looked into stress amongst students were from the Asian regions of Asia Pacific; the prevalence of stress among students in higher education institutes was recorded as between 20.6% and 70.1% (95% CI). Similar to depression and anxiety, the female gender reported higher levels of stress [1,40,44,46,48,58]. However, economic stressors and financial constraints seemed to be an avid cause of stress amongst students [1,48,59,60,61].

#### 3.2.4. Psychological Distress

Eleven studies looked into psychological distress amongst students with the prevalence ranging between 11% and 35.6% (95% CI). Lower self-esteem [12,39,54,55,62,63] was found to be one of the factors that lead to psychological distress as students who suffered from lower self-esteem and low self-efficacy were found to succumb to psychological distress. Li [64] found that students living closer to the city of Wuhan experienced higher levels of post-traumatic stress disorder compared to those who lived further away. Since Wuhan was the city where the virus broke out, Yang [65] revealed that students in Wuhan felt victimized by the COVID-19 situation and this took a negative toll on their mental health.

### 3.3. Coping Strategies Adopted by Students to Overcome Mental Health Impact

In the process of reviewing studies that were identified in Steps 2 and 3 of this scoping review, it was found that students directly or indirectly adopted different coping mechanisms to help them adapt to the pandemic situation. Table 3 displays the coping mechanisms discovered.

#### 3.3.1. Increased Screen Time

The present study found eighteen studies that showed an association between increased screen time and anxiety and depression [1,3,4,6,38,41,42,44]. Five studies found that students who were distracted by screen time also suffered from sleep deprivation [4,38,44,69]. Islam [44] found that students who live with more than five people (family or friends) around them spent more hours on the Internet. Two studies also found that students who followed online classes suffered from depression and anxiety [41,68].

#### 3.3.2. Maladaptive Coping Mechanism

Maladaptive coping mechanisms generally increase stress and anxiety [69]. Thirteen studies reported students resorting to maladaptive coping mechanisms, and they found that increased smoking was the most common (*n* = 4). A study by Kamaludin [53] found older (>25 years) male students using more maladaptive coping mechanisms. Additionally, students from different study discipline areas used different coping strategies i.e., students from the medical field (healthcare and health sciences) adopted an acceptance strategy more than students from non-medical fields [53]. Some of them may use more intense maladaptive coping strategies such suicidal ideation which is believed to have a significant relationship with sleeping problems and cigarette smoking [69].

#### 3.3.3. Social Support

Fourteen studies found students relying on a good social support systems in order to survive the pandemic. Four studies revealed that living with parents and families with sound financial backgrounds were protective factors against anxiety [11,53,70,75]. One study unveiled that students who were enrolled in campus support group programs reported having better moods and were mentally prepared to face the COVID-19 situation [75]. Additionally, a positive family cohesion was found to buffer the effect of moral disengagement on relational aggressive behavior for females [70], which goes to show that a good social support system is crucial in reducing the impact of mental health conditions.

#### 3.3.4. Religious/Spiritual

There were four studies that focused on the religious and spiritual-related coping strategies among the students. One study found that, for students, taking up meditation and an interest in religion during the pandemic lockdown helped buffer against psychological distress [47]. Kamaludin [53] identified that different race and ethnicity affected the way students embraced religious and spiritual acceptance towards the pandemic, in particular, students from Indian heritage were more prone to divert towards spiritualism. A study in Pakistan found students were turning to religion and spiritualism as a means of coping [47]. Two studies [70,74] also found students adopting rumination techniques which include meditation to help cope with the pandemic situation.

#### 3.3.5. Staying Active

Eight studies found students reverted to exercise and engagement in some form of physical activity to cope with being in lockdown [3,4,37,44,67,68,76,78]. Shailaja [68] found that students who engaged in physical activities or indulged in some form of hobby (music, cooking, gardening etc.) were able to moderate their mood better thus causing less impact on their mental health condition. In another study, Zhang [78] also discovered students who were more active reported less psychological distress.

## 4. Discussion

The systematic search for published articles related to the impact of COVID-19 on the mental health of students in the Asia Pacific region yielded 64 studies. Four mental health outcomes were identified: depression, anxiety, stress, psychological distress, and others (for example, post-traumatic stress disorder).

### 4.1. Socio Demographic Risk Factors

The current review estimates a prevalence of depression and anxiety among students during the COVID-19 pandemic of 5–72% and 7–71.5%, respectively. This was consistent with most of the studies that reported students’ psychological distress had increased during the pandemic (e.g., [1,2,3,4,5,6]). A systematic review found that the prevalence of anxiety in 17 studies was 31.9%, prevalence of depression in 14 studies was 33.7% and prevalence of stress was 29.6% [77]. This is consistent with the trend related to psychological distress during previous epidemics such as severe acute respiratory syndrome (SARS) [79]. The contagious emotion among the community led to a higher level of anxiety and other psychological distress. This was exacerbated by feelings of helplessness and uncertainty or a sense of losing control, especially with the rapidly changing and inconsistent policies given by the authorities during the pandemic. Those who followed COVID-19 related news experienced more anxiety (WHO, 2020) and misreported information about COVID-19 exacerbated students’ depressive symptoms [43].

There was a prevalence of anxiety of 20–25% among medical students prior to COVID-19 [11,68]. This finding was also lower than the prevalence of anxiety in the general student population (i.e., 38–45%), where it could have increased by two-fold [5,77]. This may be due to the higher perceived sufficiency of information on COVID’s prognosis and transmission and a broader official source of information than students in other fields that contributed to the reduction in their fears and anxiety [80].

The common factors found among students who experienced depression, stress and anxiety symptoms were gender, age, year of study and study discipline. The female gender, specifically, were more prone to the aforementioned mental health issues across studies (e.g., [3]). This is because studies have revealed that females are more perceptive, emotional, and prone to strain compared to their male counterparts [81]. Saraswathi [1] found that as opposed to males, females are more proactive in their response and awareness of the pandemic and therefore tend to succumb to depression, anxiety, and stress easily. Younger students have less resilience and self-efficacy towards handling change which leaves them vulnerable to stress and anxiety [54]. Cheng [82] also argued that younger students exposed to information through social media were more susceptible to greater anxiety. This may be due to a developmental mismatch between the mature subcortical regions that lead to risk taking, emotion dysregulation, and their higher executive functioning [83].

With respect to year of study, it has been found that students in their final year of studies [56,84] were very concerned the moment lockdown was issued particularly because there was no information on how they would be assessed in their final year and if graduation would be possible. Similar concerns were also raised by postgraduate students [34,53,68] and even more so among those postgraduate students who were on scholarships [57,84]. Dodd [84] explained how an impact on scholarship among postgraduate students had a ripple effect as they were forced to face financial constraints and relocation coupled with finding the time to complete their research at home and bearing the load of housework and childcare. All these factors added to the anxiety, depression, and stress faced by those in the final years of their education.

The last common factor identified was the study discipline students were in. This particularly affected students who were in the science fields (medical, engineering, health sciences etc.) as their lab research, clinical rotations and residency were affected due to the pandemic. Medical students also reported feeling burnout as they had to replace healthcare workers due to shortages, but they did report that the constant communication and updates from their relative schools provided them with the comfort they needed with regard to their education [57]. Furthermore, the fear of COVID-19 infection was also high among students in the medical field as they were sent to conduct swab tests and help out in hospitals during the pandemic [56].

Although a higher degree of knowledge of the disease could boost a feeling of safeness and showed a greater compliance with the proper standard operating procedure [80], most of the science field-related students were found to have more neurotic and maladaptive perfectionistic personalities as the mediator that made them more susceptible to depression and anxiety [85]. They are prone to fuse with negative evaluations of self and intense negative feelings of shame, embarrassment, and inadequacy [85]. The current COVID-19 pandemic adds to the feelings of uncertainty and loneliness in students that heighten their levels of anxiety and depression [86]. Addressing the effect of COVID-19 on this specific group of students, the urgent need for a prevention/mental health promotion programme is of uttermost importance.

### 4.2. Coping Strategies

The results of this scoping review revealed that different cohorts dealt with the pandemic by using different coping mechanisms. Males were found to be more prone to maladaptive coping mechanisms, for example, distraction leading to suicidal ideations [3,69]. Females coped more adaptively in a period of crisis by helping others in need which is consistent with their social role in society (i.e., emotionally connected with others) [61,65]. Different ethnicities also reported different means of coping; western white students were more affected by the pandemic compared to African American students [39] and resorted to substance abuse to cope. The coping strategy adopted by Asian students reflected their culture and they were more submissive to the conditions at hand and resorted to religious and spiritual means of coping [47,53].

Students who were surrounded by a good support system (family and friends) were less affected by mental health issues throughout the pandemic [75] compared to those who did not have a proper support system. Similar findings were also reported by [87] where they found students tended to act out aggression due to fear of COVID-19. However, positive family cohesion was found to buffer the effect of this aggression. Student aggression was also found to be related to the geographical location [79] of students; students in urban areas of high density felt like they were trapped as they were not able to meet with their friends and had to stay indoors most of the time due to the pandemic.

Students who were living together with more than four people under the same roof reported having higher levels of anxiety and stress [48] as they found themselves to be competing for the usage of Internet connectivity for online classes. To cope with boredom and agitated feelings of not being able to leave their premises, students found themselves spending more time online [6,41,45]. This included playing online games with their friends as a form of connection they were otherwise denied leading to sleep deprivation [1,3] and lack of physical activity [37,88].

Based on the literature in this scoping review, this paper identified four strategies that could help students to mentally cope with the pandemic. By eventually being mindful of the time spent on screen, students would be able to maintain a healthy lifestyle despite being in the pandemic situation [37,68]. Spending too much time staring into screens can not only be harmful to their mental health but also to their physical health [89]. As revealed in this scoping review, females tend to be more aware of the condition of the pandemic as they spend more time reading about it through online resources [1]. Although gathering information should reduce anxiety, Maxfield [90] revealed that information overload also contributed towards increased anxiety and stress. This showed that how to spend screen time and the quality of time spent online is the issue. If students can utilize technologies in a healthier way, for example using an online mindfulness app or counselling services [3], it helps students to alleviate their psychological issues. Despite that, having a good support system contributes to safeguarding their mental health. Studies have revealed that those who live with family tend to have less anxiety and depression compared to those living alone [49,69,72].

Psychologists have found that humans thrive better when they are surrounded by people who they can lean on [90]; therefore, having a good support system in place in times of crisis reduces the burden on their mental capitals. Exercise or participation in physical activity and pursuit of a hobby were all associated with decreased anxiety, perceived stress, and depressive symptoms [91]. Therefore, despite being in a pandemic situation, students should seek out some form of physical activity such as home workouts, gardening, learning a skill such as cooking or painting. Students who have turned to meditation during the time of pandemic reported less anxiety [47]. Burtscher [92] also suggested that a daily at-home physical activity could facilitate the routine back to university.

The last strategy would require a change in the online class system. This is especially true for disciplines that require lab work to be done. Chakraborty [56] found students were dissatisfied with webinars in replacement of lab sessions. Therefore, schools and higher education institutes should come up with another strategy to make online classes more bearable for students in the future.

Although this scoping review used a thorough search strategy to uncover published research on the impact of COVID-19 on the mental health of students in Asia Pacific, there are limitations to the study to be acknowledged. Firstly, the search was only conducted on three online databases and the papers taken into consideration were published up to March of 2021. Secondly, this paper did not look into the prevalence of anxiety, depression, stress etc. in students in the different regions of Asia Pacific as there were some papers that did not report prevalence of depression, anxiety and stress. Thirdly, the definition of students in this paper was capped to those in higher education institutes; therefore, perhaps a study in the future looking into the mental health condition of students in primary and secondary school could be looked into. It should also be noted that, although the factors identified in the current review paper might be strengthened under the COVID-19 circumstances, some of these stressors, protective factors, and risk factors have been previously identified in a non-pandemic context [93,94]. Thus, it will be beneficial to use the current paper as a baseline and compare studies again after the pandemic has ended.

## 5. Conclusions

In conclusion, the overall level of mental health issues among students in higher institutions does not appear to have increased during this COVID-19 outbreak. The present paper found that the common risk factors were younger age, being female, being in the last year of study and studying science-related fields. By reducing their screen time, using a healthier coping system, and having an increased availability of support within the family and community as well as actively engaging in beneficial activities students could alleviate general negative emotions, specifically during the pandemic.

## Figures and Tables

**Table 1:** Description of Published Research Articles Included in Scoping Review (N = 64).

Study Characteristics	Number of Studies (*n*)	Percentage (%)
Year of publication		
2020	38	59
2021	26	41
Region		
East Asia	28	44
South Asia	17	27
Southeast Asia	2	3
Oceania	2	3
North America	14	22
Multiple	1	1
Study Design		
Cross sectional	51	80
Longitudinal	6	9
^1^ Others	6	9
Not mentioned	1	2
Sample Size		
<100	1	2
100–999	38	59
1000–4999	17	27
≥5000	8	12
Target Respondents (Cohort)		
Medical students (including dental and nursing and health science)	11	17
University and college students	53	83
Scale Used		
GAD-7	18	28
PHQ	18	28
DASS-21	11	17
^2^ Others	33	51

Note: ^1^ Others: mix method/cluster sampling/snowball sampling/interviews; GAD-7: 2 Generalized Anxiety Disorder Scale; PHQ: Patient Health Questionnaire; DASS-21: Depression, Anxiety, and Stress Scale; ^2^ Others: Perceived Stress Scale; Short Mood and Feelings Questionnaire; CESD: Centre for Epidemiological Studies Depression; SDS: Self-rating Depression Scale; HADS: Hospital Anxiety and Depression Scale; Alcohol Use Disorders Identification Test (AUDIT); Zhung’s Self Rating Anxiety Scale; UCLA Loneliness Scale.

**Table 2:** Summary of studies on the mental health symptoms with corresponding factors among students.

Mental Health Symptoms	Number of Studies (*n*)	Factors Identified
Depressive Symptoms	34	Gender, age, year of study, study discipline, career concerns, academic concerns, geographical location, fear of COVID-19, race
Anxiety symptoms	39	Gender, age, year of study, study discipline, economic stressors and financial constraints, career concerns, academic concerns, geographical location, lack of outdoor activity
Stress	16	Gender, age, year of study, study discipline, economic stressors and financial constraints, social support, living condition, career concerns, academic concerns, stigmatization
Psychological Distress	11	Self-esteem, gender, study discipline, year of study, underlying medical vulnerabilities

Note: Year of Study: first year, second year, senior year etc.; Study discipline: medical, law, engineering etc.; Geographical location: rural/urban; Living condition: alone or with family/friends; Psychological distress: posttraumatic stress disorder, mood disorder etc.

**Table 3:** Summary of studies on the coping mechanisms adopted by students.

Coping Mechanism	Studies
Increased screen time	[1,3,4,6,12,38,41,42,44,49,51,52,59,60,66,67,68,69]
Maladaptive coping mechanism	[35,44,49,51,52,53,67,69,70,71,72,73,74]
Social Support	[2,11,12,34,35,39,40,43,53,68,70,75,76,77]
Religious/Spiritual	[47,53,61,74]
Staying active	[3,4,37,44,67,68,76,78]

## Data Availability

All data generated or analyzed during this study are included in this published article (and its Supplementary Information files).

## Supplementary Materials

The following supporting information can be downloaded at: https://www.mdpi.com/article/10.3390/ijerph19158894/s1, Table S1: Summarize of studies included in the review (N = 64).
