# Supplementary material for: Mental Health Risk Factors and Coping Strategies among Students in Asia Pacific during COVID-19 Pandemic—A Scoping Review
Source: Int J Environ Res Public Health. 2022 Jul 22;19(15):8894. doi: 10.3390/ijerph19158894 (PMC9329973; doi:10.3390/ijerph19158894)
Supplement: Supplementary file 1 [file ijerph-19-08894-s001.zip › ijerph-1775290-supplementary.pdf]

**Supplementary Table:**
**Summarize of studies included in the review (N =64).**

| No | Study and Country | Study Design | Sample | Assessment Tool | Prevalence Results | Coping Strategies |
|---|---|---|---|---|---|---|
| 1 | (Arima et al., 2020)<br><br>Japan | Cross sectional | *N* = 571<br>Medical Students | K-6, General Self- Efficacy Scale (GSES), Rosenberg Self- Esteem Scale (RSES) | Psychological Distress = 28.5% | Positive thinking and good mental well-being |
| 2 | (Baloch et al., 2021)<br><br>Pakistan | Cross sectional | *N* = 494<br>University Students | Zung's Self-Rating Anxiety Scale (SAS) | 58.7% - minimal anxiety<br>25.3% - moderate anxiety<br>9.1% - severe anxiety<br>6.9% - extreme anxiety | Social support from friends |
| 3 | (Brouwer et al., 2021)<br><br>USA | Co relational | *N* = 255<br>Nursing students | Kessler Screening Scale, Self-Care Assessment survey | Psychological distress:<br>(Mean = 2.33, SD = 0.79) | Positive thinking and good mental well-being |
| 4 | (Cao et al., 2020)<br><br>China | Cluster sampling | *N* = 7,143<br>Medical students | GAD7 | 0.9% - severe anxiety<br>2.7% - moderate anxiety<br>21.3% - mild anxiety. | Living with parents and households with sound financial backgrounds |
| 5 | (Chakrabothy et al., 2020)<br><br>India | Cross sectional | *N* = 168<br>Dental students | PHQ9 | 54% moderate to severe depression | NA |
| 6 | (Charles et al., 2021)<br>USA | Cross sectional | *N* = 634<br>University Students | DSM 5 Self-Rated Level 1 Cross-Cutting Symptoms Measure- Adult (CCSM); Perceived Stress Scale (PSS); Alcohol Use Disorders Identification Test (AUDIT) | psychological well-being<br>r (239) = 0.64<br>suicidal ideation<br>r (285) = 0.45<br>depression<br>r (239) = 0.58 | Racial socialization and supportive social networks |
| 7 | (Chen et al., 2020)<br><br>China | Cross sectional | *N* = 323,489<br>University Students | Regulatory Emotional Self-efficacy (RESE)<br>PHQ9 | Mean Depression = 3.47 ± 4.12.;<br>RESE = 63.95 ± 9.88.<br>7.7% of students reported depressive symptoms | Internet browsing, exercise and keeping a regular diet |
| 8 | (Conrad et al., 2021)<br><br>USA | Cross sectional | *N* = 791<br>University Students | Connor-Davidson Resilience Scale (CD-RISC-10)<br>Distress Tolerance Scale | Depression ($R^2$) = 0.049<br>Anxiety ($R^2$) = 0.076<br>PTSD ($R^2$) = 0.066 | NA |
| 9 | (Copeland et al., 2021)<br><br>USA | Sub sample of larger study | *N* = 675<br>University Students | Brief Problem Monitor (BPM/18-59)<br>Mood Scale | Mood<br>$\beta$ = 0.03, SE = 0.01, p = 0.02<br>Wellness<br>β = 0.02, SE = 0.01, p = 0.12 | Those enrolled in the campus wellness program coped better |
| 10 | (Dhar et al., 2020)<br><br>Bangladesh | Cross sectional | *N* = 15,543<br>University Students | GAD7 | 44.59% - severe anxiety<br>48.41% - moderate anxiety<br>3.82% - mild anxiety. | NA |
| 11 | (Dodd et al., 2021)<br><br>Australia | Cross sectional | *N* = 787<br>University Students | Psychological wellbeing scale, Sense of Coherence, Future Anxiety Scale, Subjective Social Status | Well-Being<br>34.7% = sufficient level of wellbeing,<br>33.8% = low wellbeing<br>31.5% = very low wellbeing | NA |
| 12 | (Faisal et al., 2021)<br><br>Bangladesh | Snowball sampling | *N* = 874<br>University Students | GAD7, CES-D Revised Scale, Mental Health Inventory-5 | 40% = moderate to severe anxiety,<br>72% = depressive symptoms<br>53% = moderate to poor mental health status | NA |
| 13 | (Feng et al., 2020)<br><br>China | Cross sectional | *N* = 1,346<br>University Students | Self-Report Altruism Scale (SRA scale), Positive and Negative Affect Schedule (PANAS), GAD7, PHQ9 | higher risk perceived = more negative affect exhibited (β = 0.16, p < .001)<br>anxiety and depression (β = 0.134, p < .001) | Positive thinking and good mental well-being |
| 14 | (Graupensperger et al., 2020) | Cross sectional | *N* = 741<br>University Students | Athletic Identity Measurement Scale, Inventory of Socially Supportive | 22.96% = moderately depressed<br>4.44% = severely depressed. | Teammate social support and connectedness |

| | | | | | | |
|---|---|---|---|---|---|---|
| | USA | | | Behaviors, Mental Health Continuum Short Form instrument, Patient-Reported Outcomes Measurement Information System | | |
| 15 | (Harries et al., 2021)<br><br>USA | Cross sectional | *N* = 741<br>Medical Students | Scale used from previously published work | Anxiety= 84.1%<br>53.5% stated adequate PPE made them feel safe returning to clinical rotations | Attending virtual town halls and daily communication updates |
| 16 | (Hasan et al., 2020)<br><br>Bangladesh | Cross sectional | *N* = 400<br>College Students | K10 | Fear of academic loss ($R^2$ = 0.434)<br>Psychological distress ($R^2$ = 0.996) | Social media usage and communicating with friends via gaming |
| 17 | (Hong et al., 2021)<br><br>China | Cross sectional | *N* = 439<br>University Students | Child and Adolescent Mindfulness Measure, Event-Related Rumination Inventory, K10 | Social media exposure (t = 1.01, p> 0.05)<br>Mindfulness (t = 0.57, p > 0.05), Rumination (t = 0.64, p > 0.05), Psychological distress (t=-0.81, p > 0.05) | Social media exposure |
| 18 | (Horita et al., 2021)<br><br>Japan | NA | *N* = 766<br>University Students | K10, CCAPS-Japanese subscale | K10 (M= 15.16, SD= 5.3)<br>CCAPS-Japanese subscale<br>Anxiety (M = 0.92, SD = 0.62)<br>Depression (M = 0.71, SD = 0.61) | NA |
| 19 | (Hossain et al., 2020)<br><br>Bangladesh | Cross sectional | *N* = 474<br>University Students | Self-rating Anxiety Scale (SAS) | 40.5% = minimal to moderate anxiety<br>2.47% = severe anxiety<br>18.1% = extreme anxiety | Seeking social support, avoidance, mental disengagement |
| 20 | (Hoyt et al., 2021)<br><br>USA | Mixed method | *N* = 707<br>University Students | GAD7, PSS | 35.6% = emotional distress<br>Anxiety n = 13 | Social support networks on campus |
| 21 | (Huckins et al., 2020)<br><br>USA | Longitudinal | *N* = 217<br>University Students | PHQ4 | Increased anxiety associated with a higher COVID-19 news ratio (P<.001) | Phone usage |
| 22 | (M. A. Islam et al., 2020)<br><br>Bangladesh | Cross sectional | *N* = 476<br>University Students | GAD7, PHQ9 | Depression = 15%<br>Anxiety = 18.1% | Living with family and engaging in exercise |
| 23 | (M. S. Islam et al., 2020)<br><br>Bangladesh | Cross sectional | *N* = 3,122<br>University Students | DASS21 | Mild depression = 76.1%<br>Moderate depression = 62.9%<br>Severe depression = 19.7%<br>Anxiety = 71.5%<br>Stress = 70.1% | Smoking tobacco, internet browsing, exercise |
| 24 | (Kamaludin et al., 2020)<br><br>Malaysia | Cross sectional | *N* = 983<br>University Students | Zung's Self-Rating Anxiety Scale (SAS) | Anxiety = 70.1%<br>Moderate anxiety = 20.4%<br>Severe anxiety = 6.6%<br>Extreme anxiety = 2.8% | Male students used more maladaptive coping |
| 25 | (Kecojevic et al., 2020)<br><br>USA | Cross sectional | *N* = 162<br>University Students | Brief Symptom Inventory, BSI-18, Perceived Stress Scale (PSS) | 64.4% = Depression<br>58.2% = Anxiety<br>53.5% = Somatization<br>20.6% = Stress | Spent more time browsing the Internet |
| 26 | (Khan et al., 2020)<br><br>Bangladesh | Cross sectional | *N* = 505<br>University Students | DASS21, IES6 | 28.5 % = stress<br>33.3% = anxiety<br>46.92% = depression | Spent more time on smartphone applications |
| 27 | (Kibbey et al., 2020)<br><br>USA | Cross sectional | *N* = 641<br>University Students | DASS21, Short Health Anxiety Inventory | 25.4 % = Depression<br>22.3% = Anxiety | NA |
| 28 | (Y. Li et al., 2021)<br><br>China | Longitudinal | *N* = 164, 101 (T1), 148, 343 (T2)<br>University Students | IES6, PHQ9, GAD7 | Acute stress<br>T1 = 34.6%; T2 = 16.4%<br>Anxiety symptoms<br>T1 = 11.4%; T2 = 14.7%<br>Depression<br>T1 = 21.6%; T2 = 26.3% | Social media exposure, smoking, alcohol consumption and exercise |

| | | | | | | |
|---|---|---|---|---|---|---|
| 29 | (W. W. Li et al., 2020)<br><br>China | Longitudinal | *N* = 178<br>University Students | DASS21 | Acute stress<br>T1 = 11.05; T2 = 7.35, T3 = 8.44<br>Anxiety<br>T1 = 9.24; T2 = 5.12, T3 = 6.69<br>Depression<br>T1 = 6.38; T2 = 5.23, T3 = 5.64 | NA |
| 30 | (X. Li et al., 2020)<br><br>China | Cross sectional | *N* = 431<br>University Students | IES6, PHQ9, GAD7 | Anxiety (3.85 ± 3.62)<br>Depression (5.73 ± 4.36) | NA |
| 31 | (X. Li et al., 2021)<br><br>China | Cross sectional | *N* = 7,747<br>University Students | Post-Traumatic Stress Disorder Symptom Checklist-Civilian version (PCL-C), PHQ-9, GAD-7 | Without ELA –<br>PTSD = 6.95%<br>Anxiety = 2.26%<br>Depression = 3.7%<br>With ELA –<br>PTSD = 8.91%<br>Anxiety = 2.6%<br>Depression = 5.26% | NA |
| 32 | (Lin et al., 2020)<br><br>China | Cross sectional | *N* = 625<br>University Students | CES-D, International Physical Activity Questionnaire - Short Form (IPAQ-SF), Chinese Sex-Role Inventory (CSRI-50) | Depression = 34.72% | Physical activity |
| 33 | (Lyons et al., 2020)<br><br>Australia | Cross sectional | *N* = 275<br>University Students | K10 | 37% = moderate psychological distress<br>26% = high psychological distress<br>11% = very high psychological distress | NA |
| 34 | (Ma et al., 2020)<br><br>China | Cross sectional | *N* = 746,217<br>University Students | IES6, PHQ9, GAD7 | Stress = 34.9%<br>Depression = 21.1%<br>Anxiety = 11.0% | Media exposure, smoking, alcohol consumption |
| 35 | (Majumdar et al., 2020)<br><br>India | Cross sectional | *N* = 325<br>University Students | CES-D | Depression = 30.3% | Increase screen time |
| 36 | (Nishimura et al., 2021)<br><br>Japan | Cross sectional | *N* = 473<br>University Students | PHQ9, GAD7 | 15.9% = Depression<br>7.2% = Anxiety | Increased screen time |
| 37 | (Nomura et al., 2021)<br><br>Japan | Cross sectional | *N* = 2,712<br>University Students | PHQ9 | 11.7% = Depressive<br>6.7% = Depressive + Suicidal | Smoking and alcohol consumption |
| 38 | (Pramukti et al., 2020)<br><br>Indonesia, Taiwan, Thailand | Cross sectional | *N* = 1,985<br>University Students | State-Trait Anxiety Inventory (STAI) | Anxiety results for:<br>Indonesian: (M = 2.33, SD = 0.48)<br>Taiwan: (M = 2.08, SD = 0.42)<br>Thai: (M = 2.55, SD = 0.43) | Increased internet browsing |
| 39 | (Rettew et al., 2021)<br><br>USA | Cross sectional | *N* = 484<br>University Students | Big Five Inventory (BFI) | Mood<br>[b = -.09 (SE = .01), p < .001, 95% CIs = -.12- -.07]<br>Wellness engagement<br>[b = -.07(SE = .01), p < .001, 95% CIs = -.10- -.04]<br>Perceived stress levels,<br>[b = -.04 (SE = .02), p = .006, 95% CIs = -.08- -.01]. | Positive thinking and good mental well-being |
| 40 | (Safa et al., 2021)<br><br>Bangladesh | Cross sectional | *N* = 425<br>Medical Students | HADS | Anxiety = 65.9%,<br>Depression = 49.9% | Social support and increased media (social media, news on TV and online newspaper etc.) exposure |
| 41 | (Salman et al., 2020)<br><br>Pakistan | Cross sectional | *N* = 1,134<br>University Students | GAD7, PHQ9 and coping strategies (Brief-COPE). | Anxiety = 34%<br>Depression = 45% | Religious/spiritual |
| 42 | (Saraswathi et al., 2020)<br><br>India | Longitudinal | *N* = 217<br>Medical Students | DASS21, Pittsburgh Sleep Quality Index | Depression = 35.5%<br>Anxiety = 33.2%<br>Stress = 24.9% | Social media exposure |

| | | | | | | |
|---|---|---|---|---|---|---|
| 43 | (Sayeed et al., 2020)<br><br>Bangladesh | Cross sectional | *N* = 589<br>University Students | DASS21 | Anxiety = 26.66%<br>Depression = 61.97%<br>Stress = 57.05% | NA |
| 44 | (Shailajah et al., 2020)<br><br>India | Cross sectional | *N* = 530<br>Medical Students | General Health Questionnaire, DASS21<br>European Health Interview<br>Survey-Quality of Life | 23.2% = depression<br>20.7% = anxiety<br>13.0% = stress | Spending time with family and pets, cultivating hobbies |
| 45 | (Son et al., 2020)<br><br>USA | Semi structured interview | *N* = 195<br>University Students | PSS | 71% = increased stress and anxiety | Denial and disengagement |
| 46 | (Sun et al., 2021)<br><br>China | Cross sectional | *N* = 1,912<br>Medical Students | IES6, PHQ9, GAD7 | 67.05% = traumatic stress<br>46.55% = depressive symptoms<br>34.73% = anxiety symptoms. | Screen media devices |
| 47 | (Tang et al., 2021)<br><br>China | Cross sectional | *N* = 2,501<br>University Students | Toronto Alexithymia Scale (TAS-20), Post-Traumatic Stress Disorder Symptom Checklist-Civilian version (PCL-C), PHQ9 | PTSD = 30.77%<br>Depression = 34.85% | NA |
| 48 | (Tasnim et al., 2020)<br><br>Bangladesh | Cross sectional | *N* = 1,979<br>University Students | DASS21 | Suicidal ideation = 12.8%<br>Depression = 63%<br>Anxiety = 63.9%<br>Stress = 58.9% | Living with family, smoking |
| 49 | (Vigo et al., 2021)<br><br>Canada | Cross sectional | *N* = 1,388<br>University Students | General Questions | 61% = symptoms of depression<br>71% = symptoms of anxiety | Living with parents |
| 50 | (Xiaomei Wang et al., 2020)<br><br>USA | Cross sectional | *N* = 2, 031<br>College Students | PHQ9, GAD7 | Depression = 48.14%,<br>Anxiety = 38.48%<br>Suicidal thoughts = 18.04% | Meditation, exercise, pursuing hobbies |
| 51 | (Xing Wang et al., 2020)<br><br>China | Cross sectional | *N* = 3, 092<br>University Students | GAD7, PSS, the Self-Rating Scale of Sleep (SRSS). | Psychological problems = 25.1%<br>Anxiety symptoms = 16.8%<br>Sleep problems = 13.5% | Frequency of news reading |
| 52 | (Wu et al., 2021)<br><br>China | Cross sectional | *N* = 11,787<br>University Students | GAD7, PHQ9 | Anxiety = 17.8%<br>Depression = 25.9% | Engaging in physical activity, increase screen time |
| 53 | (Xiao et al., 2020)<br><br>China | Cross sectional | *N* = 933<br>Medical Students | GAD7, PHQ9 | Anxiety = 17.1%,<br>Depression = 25.3% | Longer video screen time |
| 54 | (Yadav et al., 2021)<br><br>Nepal | Cross sectional | *N* = 372<br>Health science Students | GAD7, PHQ9 | Anxiety = 15.7%,<br>Depression = 10.7% | Spending hours on Internet browsing |
| 55 | (K. H. Yang et al., 2021)<br><br>China | Cross sectional | *N* = 521<br>University Students | Self-Reporting Questionnaire (SRQ-20), and Self-Rating Anxiety Scale (SAS). | 19.0% = distress,<br>31.5% = mild anxiety<br>8.1% = moderate anxiety<br>5.8% = severe anxiety | NA |
| 56 | (Y. Yang et al., 2021)<br><br>China | Cross sectional | *N* = 1,220<br>University Students | DASS21, IES6 (Revised) | Student with hearing loss<br>Mild to extremely severe stress = 37%<br>Anxiety = 22%<br>Depression = 19%<br>IES6 (Revised) = 14%<br><br>Student with normal hearing<br>Mild to extremely severe stress = 13%<br>Anxiety = 21%<br>Depression = 15%<br>IES6 (Revised) = 24% | Students with hearing loss had better mental resilience |

| | | | | | | |
|---|---|---|---|---|---|---|
| 57 | (Z. Ye et al., 2020)<br><br>China | Cross sectional | *N* = 7,800<br>University Students | Acute Stress Disorder Scale, Connor-Davidson Resilience Scale (CD-RISC), Multidimensional Scale of Perceived Social Support (MSPSS) | stressful experiences were negatively associated with resilience ($\beta$ = -0.24, $p < .001$), social support ($\beta$ = -0.20, $p < .001$), and adaptive coping strategies ($\beta$ = -0.18, $p < .001$) | NA |
| 58 | (W. Ye et al., 2020)<br><br>China | Cross sectional | *N* = 4,275<br>Medical Students and non-medical students | Perceived Stress Scales (PSS) which has been translated to Chinese (i.e., CPSS) | Stress in Medical student = 48.7%<br>Stress in Non-medical student = 36.4% | Avoidance |
| 59 | (B. Ye, D. Wu, et al., 2020)<br><br>China | Cross sectional | *N* = 841<br>University Students | Ruminative Responses Scale (RRS), Pressure Effect Scale, College Students Psychological Support Scale | Stressors of COVID (M = 2.39, SD = 0.71)<br>Rumination (M = 1.55, SD = 0.45)<br>Stress consequences (M = 1.89, SD = 0.52)<br>Psychological support (M= 3.39, SD = 0.44) | Rumination |
| 60 | (B. Ye, X. Zhou, et al., 2020)<br><br>China | Cross sectional | *N* = 1,293<br>University Students | Ruminative Response Scale (RRS) Connor-Davidson Resilience Scale (CD-RISC), Fatigue Assessment Scale, CES-D | Epidemic rumination (M = 2.97, SD = 0.54)<br>Fatigue (M = 2.59, SD = 0.50)<br>Depression (M = 1.68, SD = 0.63)<br>Resilience (M= 3.63, SD = 0.61) | Rumination |
| 61 | (Ye et al., 2021)<br><br>China | Cross sectional | *N* = 2,799<br>University Students | Moral Disengagement Scale (MDS), Family Adaptability and Cohesion Evaluation Scale, Adolescent Online Aggressive Behavior Scale | Fear of COVID = 1.88 ± 0.66<br>Moral disengagement = 1.37 ± 0.41<br>Online aggressive behavior = 1.05 ± 0.10<br>Family cohesion = 4.14 ± 0.66 | Engaging with friends online, living with family |
| 62 | (Yu et al., 2021)<br><br>China | Cross sectional | *N* = 1,681<br>University Students | CES-D, the Multi-Dimensional Scale of Perceived Social Support, the Herth Hope Index | Depression = 56.8% | Social support from family and social interactions |
| 63 | (Yunus et al., 2020)<br><br>Malaysia | Cross sectional | *N* = 1,005<br>University Students | The Oxford Happiness Inventory, Work-Family Conflict Scale, DASS21 | Stress = 22%<br>Anxiety = 34.3%<br>Depression = 37.3% | NA |
| 64 | (Yao Zhang et al., 2020)<br><br>China | Longitudinal | *N* = 66<br>University Students | International Physical Activity Questionnaire (IPAQ-S)<br>Pittsburgh Sleep Quality Index, DASS-21, Buss-Perry Aggressive Questionnaire (BPAQ), | 28.79% = stress<br>45.45% = anxiety<br>22.73% = depression emotions | Staying active and engaging in physical exercise |
